# Numerical study of transient absorption saturation in single-layer graphene for optical nanoscopy applications

**DOI:** 10.1038/s41598-024-57462-8

**Published:** 2024-04-10

**Authors:** Behjat S. Kariman, Alberto Diaspro, Paolo Bianchini

**Affiliations:** 1 Nanoscopy and NIC@IIT, Center for Human Technology, Fondazione Istituto Italiano di Tecnologia, Genoa, Italy; 2 DIFILAB, Department of Physics, University of Genoa, Genoa, Italy; 3 Present Address: Department of Physics, Politecnico di Milano, Milan, Italy

**Keywords:** Super-resolution, Single layer graphene, Ground state depletion, Transient absorption, Saturation, Nanoscale biophysics, Graphene, Optical properties and devices, Super-resolution microscopy

## Abstract

Transient absorption, or pump–probe microscopy is an absorption-based technique that can explore samples ultrafast dynamic properties and provide fluorescence-free contrast mechanisms. When applied to graphene and its derivatives, this technique exploits the graphene transient response caused by the ultrafast interband transition as the imaging contrast mechanism. The saturation of this transition is fundamental to allow for super-resolution optical far-field imaging, following the reversible saturable optical fluorescence transitions (RESOLFT) concept, although not involving fluorescence. With this aim, we propose a model to numerically compute the temporal evolution under saturation conditions of the single-layer graphene molecular states, which are involved in the transient absorption. Exploiting an algorithm based on the fourth order Runge–Kutta (RK4) method, and the density matrix approach, we numerically demonstrate that the transient absorption signal of single-layer graphene varies linearly as a function of excitation intensity until it reaches saturation. We experimentally verify this model using a custom pump–probe super-resolution microscope. The results define the intensities necessary to achieve super-resolution in a pump–probe nanoscope while studying graphene-based materials and open the possibility of predicting such a saturation process in other light-matter interactions that undergo the same transition.

## Introduction

Today’s advanced nonlinear optical imaging can solve the calls for label-free imaging with a fast frame rate (> 1 frame/s) and sub-micrometer spatial resolution in biological and material applications. Among nonlinear optical techniques, pump–probe (PP) microscopy can be considered a label-free and multi-contrast imaging modality. In this method, two pulsed laser beams are applied at different frequencies, i.e., the pump (ω_pu_) and probe (ω_pr_). They enable the exploration of other nonlinear processes^1^ including sum-frequency^2^, second-harmonic generation^3,4^, multiphoton absorption or excitation^5,6^, and stimulated Raman scattering (SRS)^7,8^. This native multimodality is implemented using a unique platform, which has tunable frequency pairs, measures the transient time of the absorption processes, and collects either the loss or gain on the transmitted probe pulse^9,10^. Such nonlinear processes, particularly transient absorption and SRS, appeal to biomedical and material applications due to their fluorescent-free nature, spatial resolution, and chemical specificity^11^. However, the spatial resolution of PP microscopy remains challenging due to the diffraction limit, as it explores optically triggered processes. Such a method can probe the molecular state dynamics related to third order nonlinearity susceptibility^12^. Under high-intensity pulsed laser excitation, transient absorption and coherent Raman processes can be saturated, and this effect can be exploited for super-resolution microscopy^10,13,14^. Moreover, since these microscopies can take advantage of near-infrared illumination wavelengths, they can be also attractive in tissue imaging^15,16^. To achieve super-resolution, we recently proposed a STED-like pump–probe approach^10,17^. Also, super-resolution SRS and coherent anti-Stokes Raman scattering (CARS) microscopy can benefit from similar methods as demonstrated in other studies^14,18^. All these approaches rely on the reversible saturable optical linear (fluorescence) transitions (RESOL(F)T) concept^19^, although fluorescence is not necessarily involved. In a scanning pump–probe microscope, two aligned beams are focused on the sample, enabling point-by-point interrogation without relying on fluorescence. While spot sizes and resolution are initially determined by diffraction, the addition of a doughnut-shaped beam that saturates a specific transition at the periphery can significantly enhance imaging resolution. When the power of this beam is increased, saturation grows, leading to enhanced resolution. However, it is crucial to carefully assess the required illumination intensity to avoid potential photodamage to the sample during imaging^20,21^. This study presents a model that estimates and predicts the power necessary to achieve saturation conditions in the transient absorption process. We specifically focus on the transient absorption occurring in single-layer graphene (SLG) when using visible/near-infrared light, which involves interband transitions^17^. Through theoretical modelling, numerical simulations, and experimental validation, we demonstrate changes in signal intensity under saturation conditions at various pump and probe intensities.

## Theoretical framework

**Figure 1:**
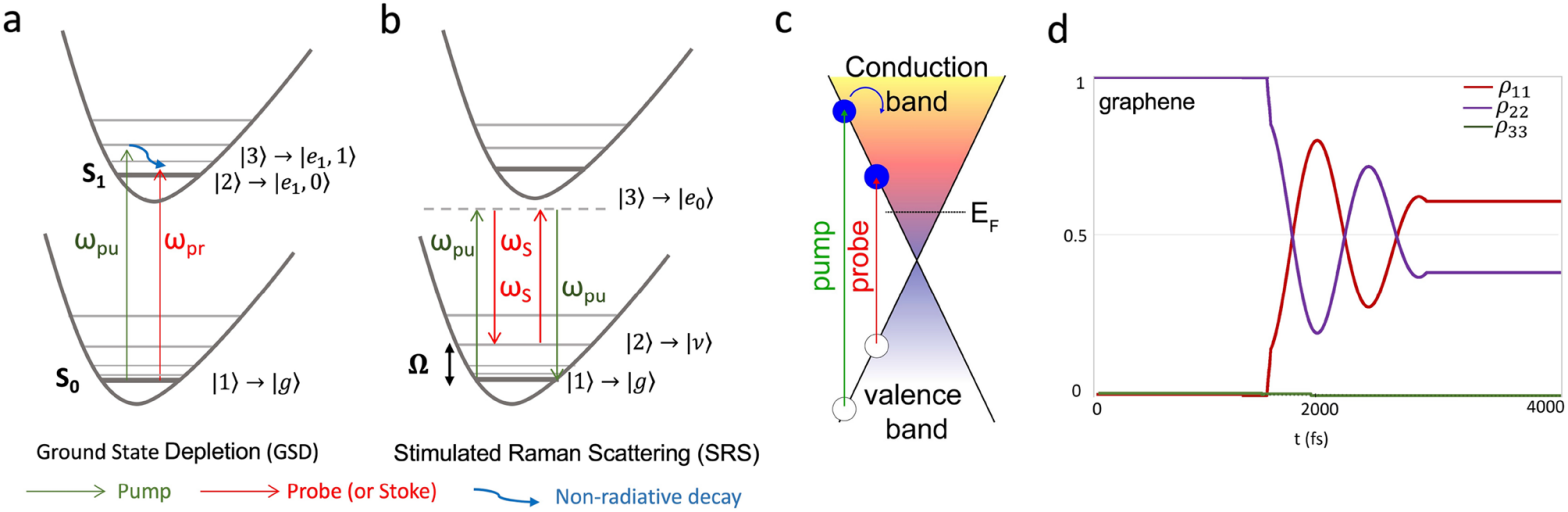
Energy level diagrams of two nonlinear optical processes, illuminating the sample with two incident beams at frequency $\omega_{pu}$ and $\omega_{pr}$ (or $\omega_{s}$). (**a**) Ground-state depletion when both the incident beams induce ground-state absorption. (**b**) Stimulated Raman scattering when $\omega_{pu} - \omega_{s}$ equals the vibrational frequency of sample, $\Omega$. (**c**) Band diagram and mechanism of TA process of graphene, E_F_ is the Fermi energy (**d**). Temporal evolution of the probability of each state in SLG considering a three levels system where τ = 0.5 ps, λ_pu_ = 805 nm, and λ_pr_ = 1030 nm.

Nonlinear optical processes can occur as a consequence of light-matter interaction through high laser intensity. The polarization ($\vec{P}$) of the material is often described as a power series expansion of the total applied optical field ($\vec{E}$). The nonlinearity results in higher order powers of $\vec{E}$ and, in particular, the third term that accounts for the third order susceptibility, $\chi^{3}$^22^, which is the focus of this work. Such a tensor is involved in many phenomena exploited in the nonlinear optics field^23^, as it implies that three optical fields interact to produce a fourth field. Although the $\chi^{3}$ interaction is thus a four-photon process; there can be up to three different input laser frequencies, but also as few as one. For example, the simultaneous absorption of two photons (2PA), the consecutive interaction of three photons followed by the generation of the final photon (THG), the absorption–emission–absorption–emission sequence (frequency mixing or CARS), and transient absorption are examples of third order nonlinear processes. In general, transient absorption involves a two-state (two energy levels) system, and it uses an ultra-short pump pulse to cause a transition from a certain state to a state at higher energy followed by an ultra-short probe pulse that records the variation^18,24^. If the first pulse produces a highly populated excited state, the absorption coefficient for the probe is reduced and the process is called ground state depletion (GSD) Fig. 1a ^10,24^. Other mechanisms, such as stimulated emission (SE), excited-state absorption (ESA), and SRS Fig. 1b ^25^, are also possible but are not the aim of this work.

In graphene, the excitation process near the Dirac point can be described by the Dirac Hamiltonian which has a linear energy dispersion dependent on the wavevector ($\varepsilon = \hbar \upsilon_{F} \left| \kappa \right|$, where $\upsilon_{F}$ = 10^6^ m/s is the Fermi velocity)^26,27^ as shown in Fig. 1c.

Upon photoexcitation by laser pulses with a wavelength in the visible/near-infrared region and duration of few hundred femtoseconds, electrons in the conduction band (ε = ћω/2) and holes in the valence band (ε = − ћω/2) are created in a non-equilibrium state while conserving momentum. Triangular warping and other nonlinear effects are considered negligible, even at visible wavelengths of up to 400 nm^28^. Considering Fermi's golden rule, we can have a complete description of graphene–light interactions^27^. Under low optical excitation where the carriers undergo fast interband decay in graphene^29^, the linear optical transmittance is dependent on the fine structure constant but independent of the excitation wavelength and the material parameter $\nu_{F}$. On the other hand, high-intensity excitation leads to significant populations of carriers in the valence band (VB) and the conduction band (CB). This results in reduced absorption of photons with the same energy (within the pulse duration), causing a bleaching effect. At the same time, the non-equilibrium carrier distributions in the CB and VB undergo ultrafast relaxation through non-dissipative carrier-carrier scattering and, to a lesser extent, carrier-optical phonon coupling^21,29^.

In this study, we consider a time-dependent uniform electric field during light interaction. To analyze its dynamic, we employ a semiclassical description derived from the Liouville-von Neumann equation with phenomenological decay term^30,31^ (Suppl. Appendix 1). Since we want to be as general as possible, we consider that nonlinear processes encompass various states such as ground state, vibration state, electronic state, and others. In the presence of intense laser excitation, overtone states may participate in transitions through nonlinear processes^31,32^. Figure 1a illustrates the specific energy level diagrams under investigation, where a sample is exposed to both pump (ω_pu_) and probe (ω_pr_). The status of population per each energy level state of the system can be presented by a density matrix description^32^ $\rho  = |{\psi}\rangle\langle{\psi} |$.

The total dynamics of the system is governed by Von Neumann equation^31,33^

1 $$\frac{\partial }{\partial t}\hat{\rho }\left( t \right) + \frac{i}{\hbar }\left[ {\hat{H}, \hat{\rho }} \right] = 0$$

Here, the notation $\left[ {\hat{H},{ }\hat{\rho }} \right]$ = $\hat{H}\hat{\rho } - \hat{\rho }{ }\hat{H}$ is called commutator^30,31^.

We take $\hat{H} = { }H_{0} + { }H_{int} + H_{r}$, where $H_{0}$ is the Hamiltonian for the matter system itself, $H_{int}$ is the Hamiltonian describing the strength of the light-matter interaction, and $H_{r}$ incorporates the relaxation process, which is considered to be time-independent. By employing the eigenfunctions of $H_{0}$ as a basis, along with other approximations and simplifications detailed in Supplementary Appendix 1, we can derive the Liouville–von Neumann equations (Eqs. 2, 3)^30^, allowing for the quantitative calculation of the temporal evolution of electron carrier populations in the system. This approach, adapted from Boyd’s textbook, semiclassically describes the light-matter interaction. It can be expressed as:^30^

2 $$\frac{{\partial \rho_{nm} }}{\partial t} = - i\omega_{nm} \rho_{{nm{ }}} - \frac{i}{\hbar }\left[ {\hat{H}_{int} ,\hat{\rho }} \right]_{nm} - { }\gamma_{nm} \rho_{nm} \quad \quad \left( {n \ne m} \right)$$

3 $$\frac{{\partial \rho_{nn} }}{\partial t} = - \frac{i}{\hbar }\left[ {\hat{H}_{int} ,\hat{\rho }} \right]_{nn} + \mathop \sum \limits_{{E_{{m \succ E_{n} }} }} {\Gamma }_{nm} \rho_{mm} - { }\mathop \sum \limits_{{E_{{m \prec E_{n} }} }} {\Gamma }_{mn} \rho_{nn}$$

these states encompass various possibilities such as ground, intermediate, vibrational, electronic, etc., denoted as $|{g}\rangle,|{i}\rangle,|{ }{\nu}\rangle ,|{e}\rangle,{ } \ldots$, respectively. The selection of 'm' and 'n' depends on the specific transition being considered. $\omega_{nm} = \upomega _{n} -\upomega _{m}$.

$\gamma_{nm}$ and ${\Gamma }_{nm}$ are decoherence and decay rates, respectively^30^.

The expression $\left[ {\hat{H}_{int} ,\hat{\rho }} \right]$ denotes the commutator, defined as $\hat{H}_{int} \hat{\rho } - \widehat{\rho }\hat{H}_{int }$ as established in prior works^30,31^.

Here, $\hat{H}_{int} { } = { }\hat{\mu } \cdot E$, represents the Hamiltonian governing light–matter interaction, where $\hat{\mu }$ is the electric dipole moment operator. This operator is derived from the absorption cross-section $\sigma_{nm}$ under the condition of zero population, with $\mu_{nm} = \langle {n}|\hat{\mu }{|}{m}\rangle$ denoting the matrix element of $\hat{\mu }$ for a system with varying levels (*n* = *m)*,

Additionally, in the context of a linearly polarized incident electric field, the relationship $\mu_{mn} = \left( {\mu_{nm} } \right)^{*}$ is valid^34,35^.

$$\mu = { }\left[ {\begin{array}{*{20}c} 0 & \cdots & {\mu_{mn} } \\ \vdots & \ddots & \vdots \\ {\mu_{nm} } & \cdots & 0 \\ \end{array} } \right],$$

and the associated density matrix is

$$\rho \left( t \right) = { }\left[ {\begin{array}{*{20}c} {\rho_{mm} \left( t \right)} & \cdots & {\rho_{mn} \left( t \right)} \\ \vdots & \ddots & \vdots \\ {\rho_{nm} \left( t \right)} & \cdots & {\rho_{nn} \left( t \right)} \\ \end{array} } \right].$$

The electric field of incident light is expressed by:

4 $$E{ } = \left[ {A_{p} \cos \left( {\omega_{pu} t} \right) + A_{pr} \cos \left( {\omega_{pr} t} \right)} \right]exp\left[ { - \frac{{2{\text{ ln}}2\left( {t - t_{0} } \right)^{2} }}{{\tau^{2} }}} \right]j,$$

where $A_{pu}$ and $A_{pr}$ denote the field amplitude of the pump and probe beams, respectively^18,30^.

Using the above Eq. (3) $A_{pu}$ and $A_{pr} { }$ are converted into the peak of intensity $I_{pu}$ and $I_{pr}$

$$I_{pu} = { }\frac{{n{\mathcal{E}}_{0} { }c{ }A_{pu}^{2} }}{2}{ },{ }I_{pr} = { }\frac{{n{\mathcal{E}}_{0} { }c{ }A_{pr}^{2} }}{2}$$

Here, *n* denotes the refractive index of the sample, *c* is the speed of light in vacuum, and ${\mathcal{E}}_{0}$ represents the vacuum permittivity^18,30^. The pulse width of both pump and probe beams are defined by $\tau_{ }$ $(\tau_{{pu{ }}} = { }\tau_{{pr{ }}} )$.

Within this quantum mechanical framework, our focus shifts towards super-resolution imaging in graphene. We synchronize the pump and probe pulses, tuning their wavelengths to 805 and 1030 nm, respectively, as these wavelengths are optimal for imaging using our custom microscope^17^. Given that the levels excited by the pump and probe pulses differs, we computed the model for a three levels system, namely the ground state in the valence band and two excited in the conduction band. However, since in graphene conduction band can be considered continuous and fast intraband relaxations occur, we expect that the populations of carriers excited by the two beams are the same, leading to an empty 'third state', Fig. 1d. In a three levels system, there could be two or more possible quantum pathways that lead to the same final state: $|{1} \rangle\to |{2}\rangle$, $|{1} \rangle\to |{2}\rangle \to |{3} \rangle\to |{2}\rangle$, $|{1}\rangle \to |{3}\rangle \to |{2}\rangle$, etc.^30,34^.

Therefore, by coupling the appropriate laser pulses, the different pathways will result in the same final state^36^. It is worth noting that the duration of the pulses has to be comparable to the relaxation time, and therefore we used it in the numerical derivation^36^. After rewriting Eqs. (2, 3) in a three levels picture, we derived all density matrix differential equations to study SLG^37,38^. We calculated the temporal evolution of carrier population in each state. We considered the pulse widths as τ = 0.5 ps, while the frequency of the pump and probe are set to be $\omega_{pu} = 2.3887{ } \times 10^{3} \;{\text{THz}}$ (805 nm) and $\omega_{pr} = 1.8278{ } \times 10^{3} \;{\text{THz}}$ (1030 nm). As shown in Fig. 1d, the probability of a third state $|{3}\rangle$ is close to zero, $6.2{ } \times { }10^{ - 14}$ Suppl. Figure 1, and thus negligible as expected. Thus, we can definitely assume the transient absorption in SLG as a two levels process where states $|{1}\rangle$ and $|{2}\rangle$ indicate the ground state, $|{g}\rangle$, and excited state, $|{e}\rangle$, are the ones involved in the process, and we modified density matrix accordingly:

$$\rho \left( t \right) = \left[ {\begin{array}{*{20}c} {\rho_{11} \left( t \right)} & {\rho_{12} \left( t \right)} \\ {\rho_{21} \left( t \right)} & {\rho_{22} \left( t \right)} \\ \end{array} } \right]$$

We consequently derived the equations^31^, from Eq. (2, 3), as

5 $$\frac{{\partial \rho_{11} }}{\partial t} = \frac{i}{2\hbar }{\upmu }_{12} {\text{E}}\left( t \right)\rho_{21} - \frac{i}{2\hbar }{\upmu }_{21} {\text{E}}^{*} \left( t \right)\rho_{12} - \frac{{\rho_{11} { }}}{{T_{1} }}{ }$$

6 $$\frac{{\partial \rho_{12} }}{\partial t} = i\omega_{21} \rho_{{12{ }}} + \frac{i}{2\hbar }{\upmu }_{12} {\text{E}}\left( t \right)\left( {\rho_{22} - \rho_{11} } \right) - \frac{{\rho_{12} }}{{T_{2} }}$$

7 $$\frac{{\partial \rho_{21} }}{\partial t} = - i\omega_{21} \rho_{{21{ }}} + \frac{i}{2\hbar }{\upmu }_{12} {\text{E}}^{*} \left( t \right)\left( {\rho_{11} - \rho_{22} } \right) - \frac{{\rho_{21} }}{{T_{2} }}$$

8 $$\frac{{\partial \rho_{22} }}{\partial t} = \frac{i}{2\hbar }{\upmu }_{21} {\text{E}}^{*} \left( t \right)\rho_{12} - \frac{i}{2\hbar }{\upmu }_{12} E\left( t \right)\rho_{21} - \frac{{\rho_{22} { }}}{{T_{1} }}$$

Hence, we set ${\Gamma }_{12} = {\Gamma }_{21} = {\Gamma }$ that we denote this quantity $\frac{1}{{T_{1} }}$, which is decay rate. Similarly, we denote the quantity $\gamma_{12} = \gamma_{21}$ as $\frac{1}{{T_{2} }}$ , which commonly is referred to decoherence rate between $|{1}\rangle$ and $|{2}\rangle$ (Suppl. Appendix 1). Then, the four Eqs. (5), (6), (7), and (8) (suppl. appendix 1 equations A5) were solved numerically using the fourth order Runge–Kutta algorithm^39^. The solution was computed over the time interval from 0 to 4 ps, with step size of 0.035 ps. Notably, the pulse was centered at $t_{0}$ = 2 ps, Suppl. Figure 1c. We specifically focused on t = 4 ps, a time point by which the light-matter interaction is nearly complete^18,40^. The algorithm, written in Python (available upon request), calculates the temporal evolution of carriers populating different interband levels of single-layer graphene.

## Materials and methods

### Single-layer graphene (SLG)

Sample was purchased from Graphene Supermarket (Graphene Laboratories Inc., Calverton, NY). It consists of a monolayer graphene film grown by chemical vapor deposition (CVD) processing onto copper foil and then transferred onto a 0.17 mm thick glass substrate. This slide with the deposited SLG was mounted on a 0.17 mm cover glass to be placed under a water immersion objective. The graphene film is mostly continuous, with occasional holes and cracks, and has a polycrystalline structure made of grains with different crystallographic orientations. The supplier reports the sample transmission to be above 97% in the visible spectral window, which makes it suitable to collect the signal efficiently in transmission.

### Pump–probe nanoscopy

**Figure 2:**
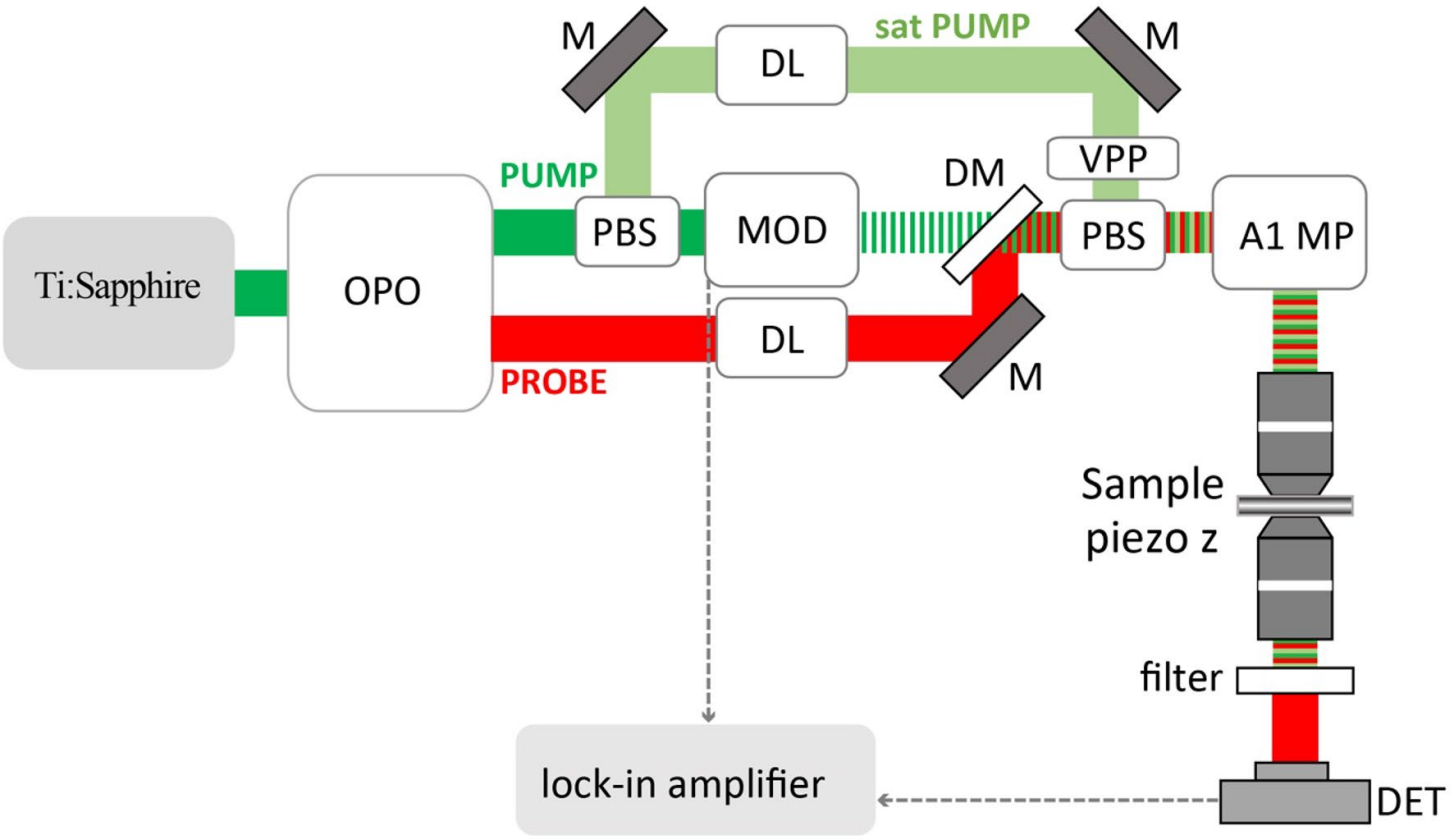
Scheme of the pump–probe nanoscope setup. *OPO* optical parametric oscillator, *EOM* electro-optical modulator, *M* mirror, *DM* dichroic mirror, *VPP* vortex phase plate, *PBS* polarizing beam splitter, *DL* delay line, *A1 MP* Confocal and multiphoton laser scanning Nikon microscope (A1 MPr Nikon microscope, Nikon Instruments, Yokohama, Japan), *DET* detector.

In this work, pump–probe, and saturated pump–probe images were performed using a custom-built near-infrared pump–probe nanoscope. Figure 2 shows the schematic illustration of the pump–probe microscopy system. Our pump–probe nanoscope consists of a tunable mode-locked femtosecond pulsed Ti: sapphire laser (680–1080 nm, 80 MHz, 140 fs, Chameleon Ultra II, coherent), a commercial laser scanning confocal Nikon A1 MP microscope, and the optical items depicted in Fig. 2 that allow realizing the three optical beams configuration. Two femtosecond pulsed laser beams generated by an OPO (Coherent Inc., Santa Clara, CA, USA) pumped by a laser source are used as the pump (tunable in the range 740–880 nm) and probe (tunable in the range 1000–1600 nm), respectively. The pump beam intensity is modulated at 1 MHz through an electro-optical modulator EOM (LM 0202, Qioptiq, Goettingen, Germany). A digital delay generator controls and generates the electronic synchronization between the pulse rate and the modulation (DG645, Stanford Research Systems, Sunnyvale, CA). A motorized delay line (M-521.PD, Physik Instrument, Karlsruhe, Germany), equipped with a retroreflector (UBBR2.5 − 2I, Newport Corp., Irvine, CA) delays up to 1.3 ns with 3 fs accuracy. For most of the experiments (imaging and saturation measurements), the pump beam’s pulse and the probe beam's pulse have been synchronized. Also, the two beams (pump and probe) are spatially combined and overlapped through a dichroic mirror and delivered into a Nikon Confocal A1 (multiphoton) scanning microscope and are focused onto the sample with an objective lens (40 × 1.15 NA, water immersion). In the saturated case, the pump beam is taken before the EOM with a polarizing beam splitter (PBS, Thorlabs, Newton, NJ). Then the beam passes through another motorized delay line (LTS150/M, Thorlabs, Newton, NJ, USA) with a delay of 1 ns with 16 fs accuracy used to adjust its temporal alignment connecting pump and probe pulses. The beam passes through a vortex phase plate (VPP-1a, RPC Photonics, Rochester, NY, USA) to create a doughnut shape coupled with the other beam by a polarizing beam splitter (PBS, Thorlabs, Newton, NJ). The probe signal is collected in transmission by an objective (20 × 0.67 NA, water immersion) filtered by two long-pass filters (RG850, Schott, Mainz, Germany, and FEL1000, Thorlabs, Newton, NJ) to eliminate pump light and detected by an amplified InGaAs detector (PDA20CS, Thorlabs, Newton, NJ, USA). The detector is connected to a lock-in amplifier (Model SR844 RF, Stanford Research System, Sunnyvale, CA) to demodulate the probe signal and extract the pump–probe signal in the module channel (R) at the pump modulation frequency. The controller for non-descanned detectors of the Nikon A1MP acquires the demodulated probe signal, and images are collected using NIS-Element Advanced Research software (Nikon Instruments, Yokohama, Japan).

## Result and discussion

Single-layer graphene (SLG) has a high nonlinear third order $\chi^{3}$ susceptibility, which enables harmonic generation, transient absorption, and frequency mixing. Such a property is due to the optical resonance among interband electronic transitions^41–44^. To estimate the energy transferred to the upper level in SLG, we quantitatively calculated the population temporal evolution for each molecular state at pump = 805 nm, probe = 1030 nm, and pulse width = 500 fs. As we discussed in appendix, in this model, off-diagonal elements of the electric dipole moment matrix have the same value, set to $\mu_{mn} = 0.196 e \cdot nm$ when $\left( {n \ne m} \right)$, which are derived from the absorption cross-section $\sigma_{ij}$^32^, while all diagonal elements are set to zero (Suppl. Appendix 1). The refractive index of SLG was assumed to be n = 2.58^41^. The decay of off-diagonal elements of density matrix $\rho_{nm}$ is caused by decoherency between two-state, which the decoherent rate can be obtained with bandwidth of absorption spectrum^10,18,32^. Also, the decoherent rate between vibrational and ground state can be estimated from bandwidth of the spontaneous Raman spectrum^18,33,39^ We measured the decoherent rate between ground and upper excited state by time-resolved spectrum^18^ of pump–probe (Fig. 4c), and we set to be $\gamma_{nm} { } = { }0$ for (n = m), and $\gamma_{nm} { } = { }1.824\;{\text{ps}}^{ - 1}$^10,39^ when (m ≠ n) (Suppl. Appendix 1), and $\left( {\hbar \left( {\omega_{pu} + \omega_{pr} } \right) = 2744 meV} \right)$. The interband relaxation time of the carriers is about 150 fs while the following interband electron–hole recombination has a time decay of about 2 ps^45^ Therefore, in our simulations, we considered different values of ${\Gamma } = 0.01, 0.1,\;{\text{and}}\;0.11 \;{\text{ps}}^{ - 1}$(Table 1).

**Table 1:** It shows the values of the intensities required to reach saturation when $\rho_{11} { } = { }\rho_{22} { } \approx { }1/2$ for different values of ${\Gamma }$.

	$\Gamma = 0.01$		$\Gamma = 0.1$		$\Gamma =$ 0.11	
	$I_{pu}$	$I_{pr}$	$I_{pu}$	$I_{pr}$	$I_{pu}$	$I_{pr}$
	$$\left( {{\raise0.7ex\hbox{${{\text{GW}}}$} \!\mathord{\left/ {\vphantom {{{\text{GW}}} {{\text{cm}}^{2} }}}\right.\kern-0pt} \!\lower0.7ex\hbox{${{\text{cm}}^{2} }$}}} \right)$$	$$\left( {{\raise0.7ex\hbox{${{\text{GW}}}$} \!\mathord{\left/ {\vphantom {{{\text{GW}}} {{\text{cm}}^{2} }}}\right.\kern-0pt} \!\lower0.7ex\hbox{${{\text{cm}}^{2} }$}}} \right)$$	$$\left( {{\raise0.7ex\hbox{${{\text{GW}}}$} \!\mathord{\left/ {\vphantom {{{\text{GW}}} {{\text{cm}}^{2} }}}\right.\kern-0pt} \!\lower0.7ex\hbox{${{\text{cm}}^{2} }$}}} \right)$$	$$\left( {{\raise0.7ex\hbox{${{\text{GW}}}$} \!\mathord{\left/ {\vphantom {{{\text{GW}}} {{\text{cm}}^{2} }}}\right.\kern-0pt} \!\lower0.7ex\hbox{${{\text{cm}}^{2} }$}}} \right)$$	$$\left( {{\raise0.7ex\hbox{${{\text{GW}}}$} \!\mathord{\left/ {\vphantom {{{\text{GW}}} {{\text{cm}}^{2} }}}\right.\kern-0pt} \!\lower0.7ex\hbox{${{\text{cm}}^{2} }$}}} \right)$$	$$\left( {{\raise0.7ex\hbox{${{\text{GW}}}$} \!\mathord{\left/ {\vphantom {{{\text{GW}}} {{\text{cm}}^{2} }}}\right.\kern-0pt} \!\lower0.7ex\hbox{${{\text{cm}}^{2} }$}}} \right)$$
$\frac{{\rho_{11} }}{{\rho_{22} }} \approx 1$	109	10	140	10	130	10
$\rho_{11} = \rho_{22} \approx 1/2$	$\lambda_{pu}$ (nm) = 805	$\lambda_{pr}$ (nm) = 1030				

Then, the algorithm was computed from 0 to 4 ps to completely simulate the entire light-matter interaction, which is completed in < 4 ps. In this model, we made the simplified assumption that all the carriers are in the ground state ($\rho_{gg}$ = 1); after light interaction they undergo the electronic transition, $|{g} \rangle\to |{e}\rangle$, and the population in the ground state, $|{g}\rangle$ decreases while the population in the upper excited state, $|{e}\rangle$, increases until saturation occurs. We calculate $\rho_{ee}$ and $\rho_{gg}$ at different values of $I_{pu}$ and $I_{pr}$ to estimate the required laser intensity to reach the complete depletion of the ground state. In the PP process, the energy transferred to excited states can also be assessed by $E_{{exc{ }}} = { }\rho_{nn} |_{t = 4ps} \hbar \omega_{nm}$, where the n and m terms describe all the possible lower and upper excited states, respectively. If we assume that the number of annihilated photons for both the pump and probe are equal, then the number of photons lost per molecule in the PP process is $\frac{{E_{{exc{ }}} }}{{\hbar { }\left( {\omega_{pu} + \omega_{pu} } \right)}}$. Typically, the pump–probe signal, ($\Delta T$), exhibits a weak nonlinearity, and its intensity will vary proportionally to the analyte concentration $N_{0}$, and the product of the pump and probe intensities $I_{pu}$ and $I_{pr}$, respectively, $\Delta T \propto { }N_{0} { }I_{pu} I_{pr}$^46^. In the case of the GSD mechanism, the absorption of an intense pump beam causes a population inversion from the ground to the excited state, and consequently the decrease of the absorption coefficient. Thus, a probe beam in resonance with the absorption transition will exhibit a transmission increase. Such a behavior can be derived from the Lambert–Beer relation^47^:

$${\Delta }T = { } - \smallint \frac{{N_{0} \sigma_{pu} \sigma_{pr} I_{pu} I_{pr} exp\left( {{\raise0.7ex\hbox{${ - \Delta t}$} \!\mathord{\left/ {\vphantom {{ - \Delta t} \tau }}\right.\kern-0pt} \!\lower0.7ex\hbox{$\tau $}}} \right)}}{{\hbar \omega_{pu} }}d\tau$$

where $N_{0}$ is the molecular concentration of the ground state, $\sigma_{pu}$ and $\sigma_{pr}$ are the linear absorption cross-sections of the ground state for the pump and probe beams, respectively, $\Delta t$ is the time delay between pump and probe pulses, and τ is the lifetime of the excited state. When $\Delta t = 0$ the strongest signal is achieved which exponentially decays at longer $\Delta t$, reflecting the characteristic relaxation time τ of the excited state^47^.

**Figure 3:**
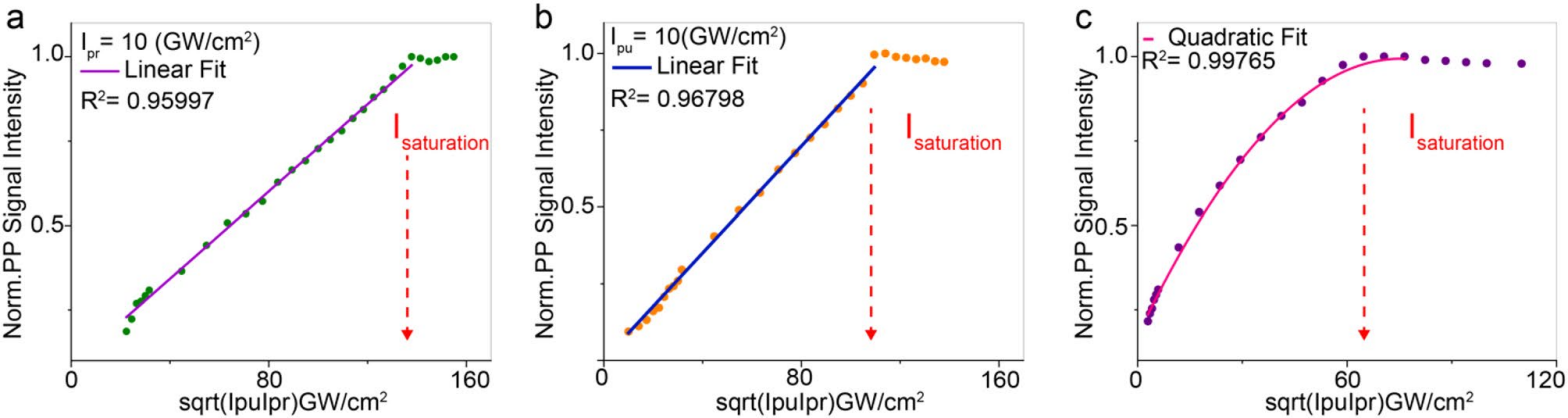
Numerical simulations of PP signal in SLG as function of the illumination powers. (**a**, **b**) The PP signal as a function $\sqrt {I_{pu} I_{pr} }$, for $\lambda_{eg} = 805{\text{nm}}$, $\tau = 500fs$, while $I_{pr}$ is kept constant in (10 ${\text{GW/cm}}^{2}$) (**a**), and $I_{pu}$ is constant in (10 ${\text{GW/cm}}^{2}$) (**b**). (**c**) PP signal of SLG as $\sqrt {I_{pu} I_{pr} }$, for $\lambda_{eg} = 805\;{\text{nm}}$, $\tau = 500\;{\text{fs}}$, while depends on total intensities ($I_{pu} + I_{pr}$), where the initial intensities were assumed to be $I_{pu}$ = $I_{pr}$ = $2.94\;{\text{GW/cm}}^{2}$. Saturation intensities are defined in (**a**–**c**). R-square is the coefficient of determination, a value between 0 and 1 that statistically qualify the regression.

**Figure 4:**
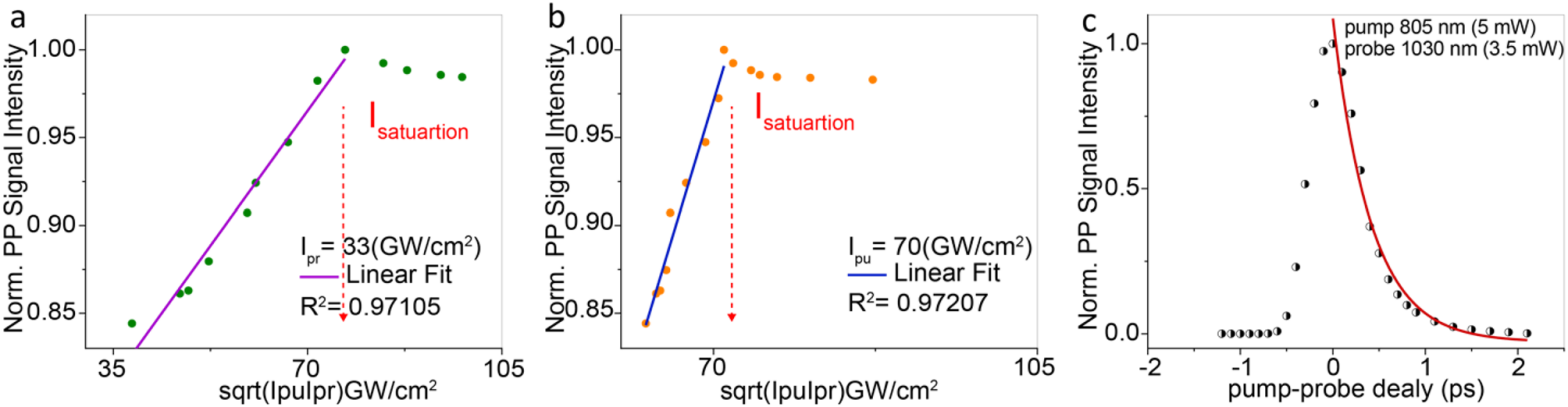
PP microscopy experiments on SLG. The PP signal versus $\sqrt {{\text{I}}_{{{\text{pu}}}} {\text{I}}_{{{\text{pr}}}} }$, for ${\uplambda }_{{{\text{pu}}}} { } = 805\,{\text{nm}}$, ${\uplambda }_{{{\text{pr}}}} { } = { }1030\,{\text{nm}}$, while ${\text{I}}_{{{\text{pr}}}}$ is kept constant at (33 ${\text{GW/cm}}^{2}$) (**a**), and ${\text{I}}_{{{\text{pu}}}}$ is constant at $\left( {70\;{\text{GW/cm}}^{2} } \right)$ (**b**). (**c**) Time-resolved spectra of SLG defects obtained at different delays of the probe pulses with respect to pump pulses. The pump–probe signal was fitted with an exponential decay which retrieved a fast component with (1.38 ± 0.04) ps for lifetime. The R-square is the coefficient of determination, a value between 0 and 1 that statistically qualifies the regression.

To study how the population changes at the interband level as a function of beam intensity, we assumed the pump power, $I_{pu}$, increases while probe power, $I_{pr}$, remains constant and vice-versa. Panels a and b in Fig. 3 show that the pump–probe signal intensity has a linear dependency in both conditions up to a specific limit where the initial intensities were assumed to be $I_{pu}$ = $I_{pr}$ = $10 \;{\text{GW/cm}}^{2}$. Moreover, we calculated the pump–probe signal intensity under different total powers ($I_{pp}$ = $I_{pu}$ + $I_{pr}$), which fits a quadratic polynomial behavior, plotted in (Fig. 3c). The initial intensities were assumed to be $I_{pu}$ = $I_{pr}$ = $2.94 \;{\text{GW/cm}}^{2}$. It is worth noting that to achieve absorption saturation our simulation uses a slightly higher intensity. The theoretical findings can be essential to quantitatively predict the required excitation power to achieve saturation, thus resolving the parameters to maximize super-resolution imaging performance. In fact, the optical resolution, d, defined by Abbe’s law as the shortest distance between two points that can be distinguished as separate units, is reduced by a factor that depends on saturation and can be written in the following equation:

9 $$d^{\prime} \cong \frac{{\text{d}}}{{\sqrt {1 + {\text{c}}\frac{{\text{I}}}{{I_{s} }}} }}$$

where ${\text{c}} = \frac{ln2}{2}$, $I_{s}$ is the saturation factor defined as the intensity necessary to force 50% depopulation of the ground state, and ${\text{I}}$ is the intensity distributed in a doughnut shaped beam at the periphery of the pump and probe beams (sat-PUMP in Fig. 2)^48^. In this context, we acquired pump–probe images of SLG using our custom-built near-infrared pump–probe nanoscope (Fig. 2) to experimentally validate our numerical simulation, verifying the real effect of power changes. The pump–probe intensity signal was acquired sequentially while imaging SLG and increasing $I_{pu}$ and $I_{pr}$ respectively. Figure 4a shows PP signal vs $\sqrt {I_{pu} I_{pr} }$ increasing $I_{pu}$ from ($44\;{\text{ to}}\;287\;{\text{ GW/cm}}^{2}$), while $I_{pr}$ is kept constant at ($33 \;{\text{GW/cm}}^{2}$), and Fig. 4b shows the same but increasing $I_{pr}$ from $55\;{\text{ to}}\; 107 \;{\text{GW/cm}}^{2}$, while $I_{pu}$ is constant at (70 ${\text{GW/cm}}^{2}$). Also, we measured the time decay of the transition (Fig. 4c) which resulted in line with the literature^10^.

The theoretical (Fig. 3a, b) and experimental (Fig. 4a, b) results show that the intensities required to reach saturation are approximately $I_{sat} \approx$ $3I_{pu} , 3I_{pr}$ in simulation, and $I_{sat} \approx$ $2I_{pu} , 2I_{pr}$ experimentally. These finding can be key to quantitatively predicting the required excitation power to achieve saturation, allowing optimisation of the parameters to maximize the performance of super-resolution imaging. Our numerical simulations and experimental data are in good agreement, showing that we can efficiently achieve super-resolution while imaging SLG.

**Figure 5:**
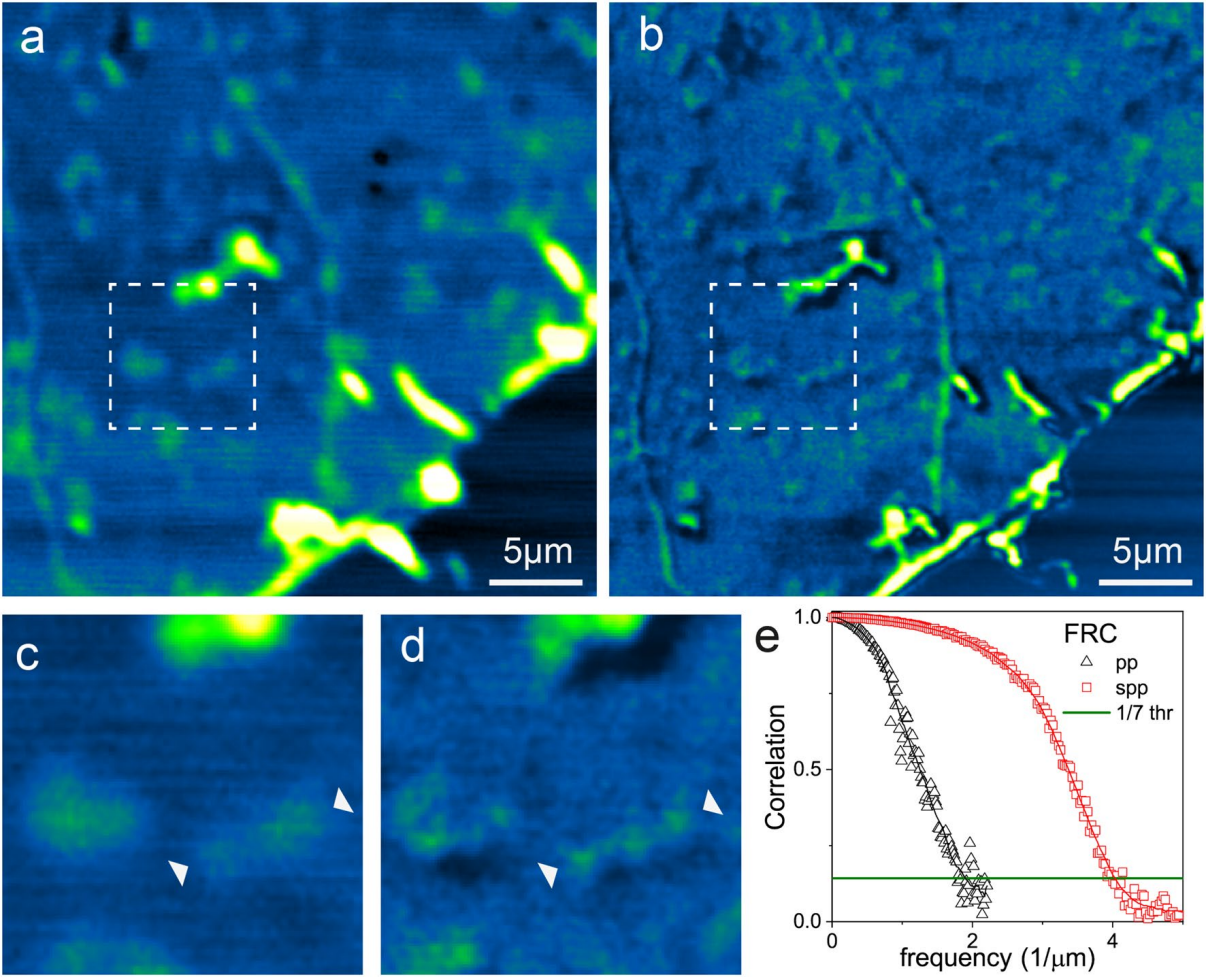
Normalized pump–probe (pp) (**a**) and saturated pump–probe (spp) (**b**) images of SLG foldings and defects. (**c**, **d**) Zoomed region of (**a**, **b**) respectively the arrows highlight the resolution improved obtained in spp. (**e**) is the plot of the Fourier Ring Correlation (FRC) of the two images: (**a**) in black and (**b**) in red. The FRC has been obtained using two sequential images, (**a**) and (**b**) are the sum of such images. FRC calculation has been carried on using carried out by the FRC_plugin of ImageJ available from the PTBIOP Update Site (https://www.epfl.ch/research/facilities/ptbiop/). Therefore, the lateral resolution in (**a**, **b**) is better than 530 nm and 250 nm, respectively. The pump and probe wavelengths used are $\lambda_{pu } = 805 \;{\text{nm}},$ $\lambda_{pr} = 1030\;{\text{nm}}$, respectively.

In our PP microscope, the resolution is improved by using an unmodulated doughnut-shaped pump beam overlapped with the other two conventional beams, all focused on the sample. The beam is shaped using a vortex phase plate, and its polarization is made circular with two retarder plates, i.e., a half-and a quarter-wave plate^49^. A dedicated optical delay line is used to synchronize the pump and probe pulses, and all the beams are synchronized at time zero to maximize the signal-to-noise ratio. The doughnut-shaped pump beam, realized with the vortex phase plate (VPP in Fig. 2), saturating the depletion of the ground state, can avoid the probe absorption at the rim of the focal spot, leading to STED-like super-resolution^10,13^. We obtained a resolution improvement by tuning the wavelengths and power in the following manner: pump = 805 nm with 4 mW, probe = 1030 nm with 5 mW, and saturation-pump = 805 nm with 11 mW. To perform super-resolution imaging, we set the power of unmodulated saturation pump to $I_{satpump} \approx 2I_{pr}$. As shown in Fig. 5, panels a and b, we acquired unsaturated and saturated pump–probe images of SLG after defining the conditions to achieve ground state depletion saturation and, therefore, super-resolution. From the insets in Fig. 5c, d, we highlight some details that are better resolved in saturated pump–probe condition. The spatial resolution is quantitatively measured by Fourier Ring Correlation^50,51^, Fig. 5e, resulting in being better than ${\uplambda }/4$ and in agreement with the literature^10^, another example at lower saturation intensity is presented in suppl. Figure 2.

It is worth noting that such an improvement is not the best achievable resolution but is what can be already obtained at a power above the one we predicted saturation happens. Since saturation occurs, the resolution will improve at higher powers.

## Conclusions

We have proposed a theoretical model and an experimental study on the temporal behavior of single-layer graphene absorption states under strong illumination in the NIR spectral window. We numerically and experimentally presented how the pump–probe signals of SLG depend upon increasing illumination intensity varying ${I}_{pu}$ or ${I}_{pr}$. We successfully calculated the required peak intensity to achieve the saturation of the transient absorption process in graphene, and validated our simulation with experimental data and super-resolution imaging. Thus, our theoretical model can have the potential to predict the light intensity required to reach nanoscale optical resolutions on a diverse range of materials.

### Supplementary Information

Supplementary Information.

## Data Availability

The datasets used and/or analysed during the current study available from the corresponding author on reasonable request.
