# Supplementary material for: Numerical study of transient absorption saturation in single-layer graphene for optical nanoscopy applications
Source: Sci Rep. 2024 Apr 10;14:8392. doi: 10.1038/s41598-024-57462-8 (PMC11350070; doi:10.1038/s41598-024-57462-8)
Supplement: Supplementary file 1 — Supplementary Information. [file 41598_2024_57462_MOESM1_ESM.docx]

*Supplementary materials*

Numerical study of transient absorption saturation in single-layer graphene for optical nanoscopy applications

Behjat S. Kariman^1,2 †^, Alberto Diaspro^1,2^, Paolo Bianchini^1,2 *^

^1^ Nanoscopy and NIC@IIT, Center for Human Technology, Fondazione Istituto Italiano di Tecnologia, Genoa, Italy

^2^ DIFILAB, Department of Physics, University of Genoa, Genoa, Italy

^†^present address: Department of Physics, Politecnico di Milano, Milan, Italy

***** Correspondence: Paolo Bianchini, [paolo.bianchini@iit.it](mailto:paolo.bianchini@iit.it)

**Supplementary Materials**


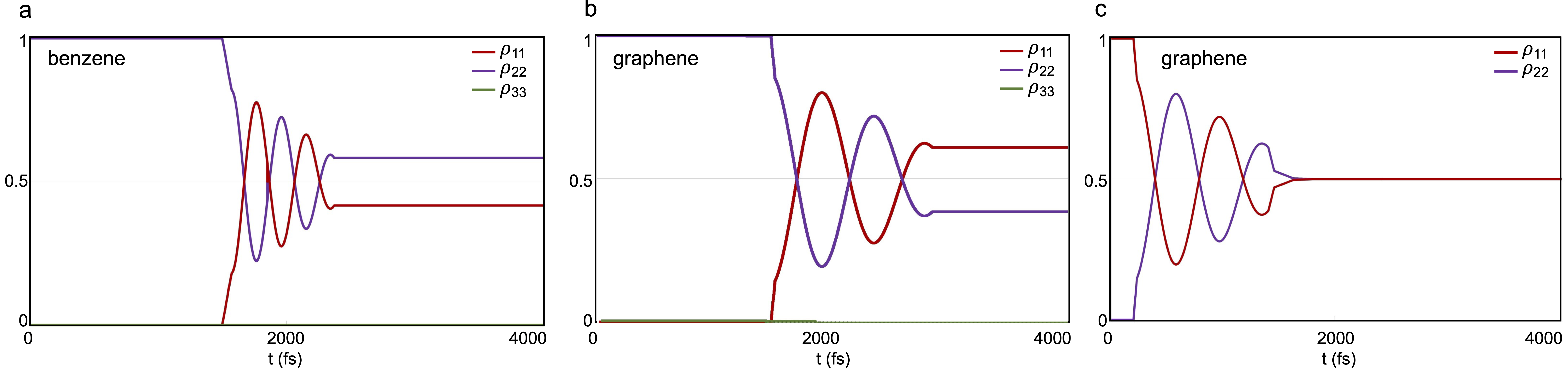


**Supplementary Figure 1**

Temporal evolution of the probability for each state in benzene (a) and in single-layer graphene (b and c) under saturation conditions. The states include ground, vibration, and electronic states depending on the type of light-matter interaction process involved. The illumination scheme is the same in all cases, and the sample is irradiated by a pump and a probe (or Stokes) beam. For benzene (a), where SRS occurs during the entire light-molecule interaction, the population probability of the third state is small ($\rho_{33} \approx0.0079$) ^1^, leading to a little absorption of the third state, i.e., the first electronic excited state. We considered the pulse widths as 𝜏 = 2 ps, while the electronic excitation frequency was set to be, $\omega_{ge} = 7.2498 \times{10}^{3} THz$ (260 nm). The frequency of the pump and probe (Stokes) are set to be $\omega_{pu} = 2.5422 {\times10}^{3} THz$ (741 nm) and $\omega_{pr} = 2.3550 {\times10}^{3} THz$ (800 nm). The results are in agreement with the previous results published by Gong et al. ^2^.

In the case of SLG, transient absorption occurs^3,4^, and we show the temporal evolution calculated considering three (b) and two states (c). In (b), the states are the ground state, $\rho_{11}$, the electronic excited state, $\rho_{22}$, and a higher electronic excited state, $\rho_{33}$. We considered pulse width as $\tau= 0.5 ps$, $\lambda_{\mathrm{pu}} = 805 nm$, and $\lambda_{pr} = 1030 nm$, $I_{pu} = I_{pr} = 110\frac{GW}{{cm}^{2}}$. The population probability of such a third state in graphene is five orders of magnitude smaller than benzene and thus negligible as expected. In (c) the states are the ground state, $\rho_{11}$, and electronic excited state $\rho_{22}.$The algorithm was calculated from 0 to 4 ps with 0.035 fs step size, while $\Gamma$=0.11. Furthermore, we defined the condition of achieving equilibrium as the probability of being found in the excited and ground state set to 1/2, which simplifies the normalisation condition $\left| \rho_{11}\left( t \right) \right| + \left| \rho_{22}\left( t \right) \right| = 1$ . Since the equilibrium occurs after 2 ps for higher excitation power, the center of the pulses was set at $t_{0}$= 2 ps to simplify the calculation and show the oscillations.


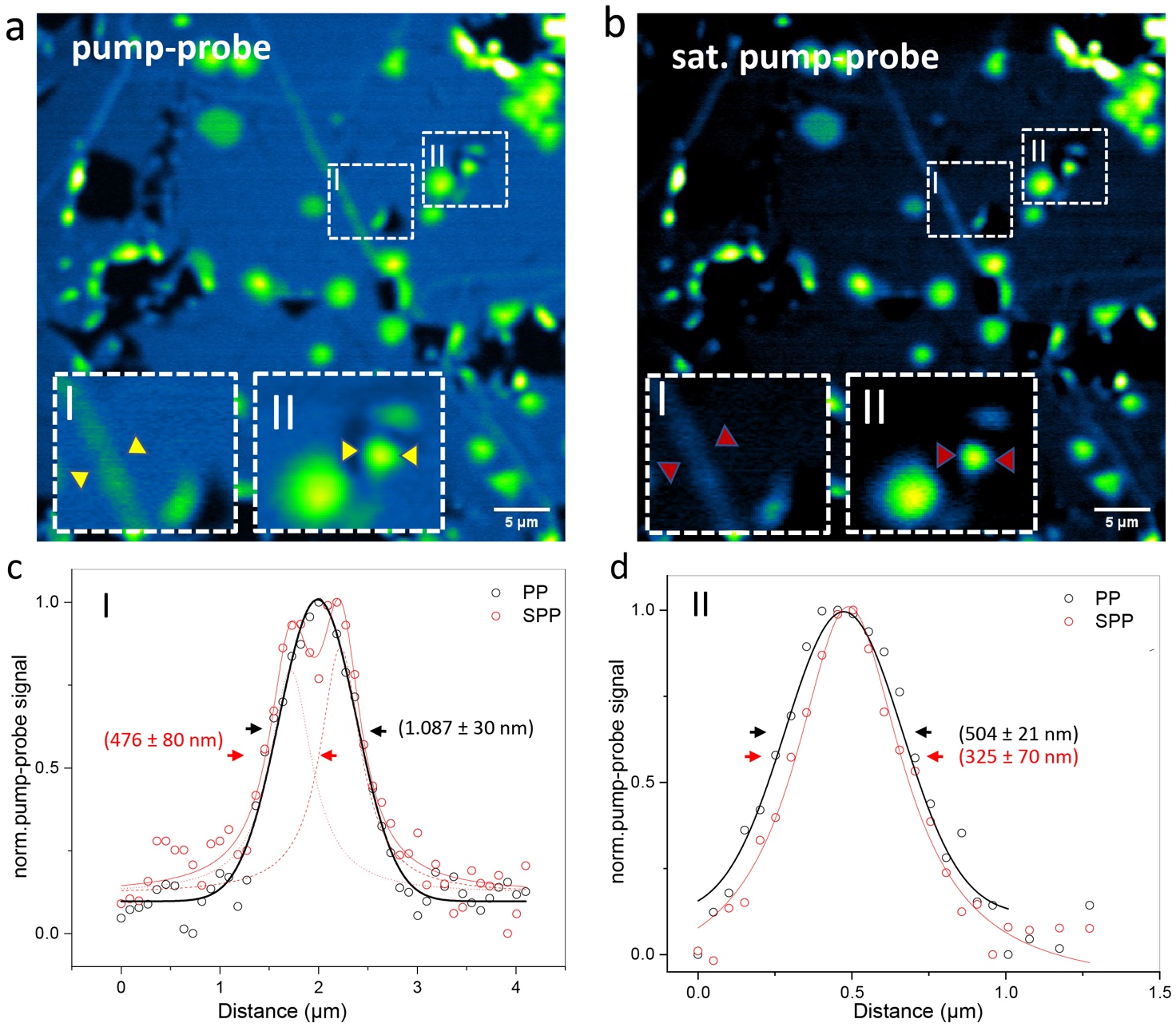


**Supplementary Figure 2**

Normalised pump-probe (a) and saturated pump-probe (b) images of SLG foldings and defects. Zoomed regions I and II are also presented (panels c and d, respectively), and line profiles across the arrows are shown as black dots (non-saturated case, PP) and red dots (saturated case, SPP). The Gaussian fits of the non-saturated data are shown as black solid lines, while Lorentzian double-peak (c, I) and single-peak (d, II) fits of the saturated data are shown as solid red lines. The dotted and dashed red lines in graph I highlight the single Lorentz peaks retrieved from the analysis. The obtained resolution is marked as FWHM of the fitted curves in the graphs. The resolution improvement was obtained by tuning the wavelengths and power in the following manner: pump = 805 nm with 2.9 mW, probe = 1030 nm with 3.9 mW, and saturation-pump = 805 nm with 7.9 mW.

**Supplementary Appendix 1**

Density matrix.

For a pure state, the density matrix of a two-level system can be described by $\hat{\rho}= \left. |\psi\right\rangle\left\langle\psi| \right.= \left( \begin{matrix} \left| C_{1} \right|^{2} & C_{2}^{*}C_{1} \\ C_{1}^{*}C_{2} & \left| C_{2} \right|^{2} \end{matrix} \right)$, which the diagonal elements yield the populations, whereas the off-diagonal elements describe the coherent motion of dipole momentum^5^.

For general mixed state where we have an ensemble of particles (e.g., photoexcited charge carriers, ...), the probability of particles of different states can be described by ^5,6^

$\hat{\rho}= \sum_{n} p_{n}= \left. |\psi_{n} \right\rangle\left\langle\psi_{n}| \right.$ with $0\leq p_{n}\leq1, \sum_{n} p_{n}=1$

The total dynamics of the system is governed by Von Neumann equation^5,6^

$\frac{\partial}{\partial t}\hat{\rho}\left( t \right)+ \frac{i}{\hbar}\left[ \hat{H}, \hat{\rho} \right]=0$

However, to analyse problems with time-dependent perturbation, it is sufficient to determine the time dependence of $\rho$. Taking the time derivation of $\rho$ as defined in

$\hat{\rho}= \sum_{n} p_{n}= \left. |\psi_{n} \right\rangle\left\langle\psi_{n}| \right.$,

and requiring that all of the $\left. \mathbf{|}\boldsymbol{\psi}_{\boldsymbol{n}} \right\rangle$ satisfy Schrodinger’s equation, thus we have^5^,

$\boldsymbol{i}\boldsymbol{\hbar}\dot{\boldsymbol{\rho}}\mathbf{=}\sum_{\boldsymbol{n}} \boldsymbol{p}_{\boldsymbol{n}}\left( \boldsymbol{H} \left. \mathbf{|}\boldsymbol{\psi}_{\boldsymbol{n}} \right\rangle\left\langle\boldsymbol{\psi}_{\boldsymbol{n}}\mathbf{|-}\left. \mathbf{|}\boldsymbol{\psi}_{\boldsymbol{n}} \right\rangle\left\langle\boldsymbol{\psi}_{\boldsymbol{n}}\mathbf{| H} \right. \right. \right)\mathbf{=}\left[ \boldsymbol{H}\mathbf{,}\boldsymbol{\rho} \right]$ (A 1)

Where the notation $\left[ \hat{H}, \hat{\rho} \right]$ = $\hat{H}\hat{\rho}-\hat{\rho} \hat{H}$ is called commutator ^5,7^.

We take $\hat{H}= H_{0}+ H_{int}+H_{r}$, where $H_{0}$ is the Hamiltonian for the matter system itself ; $H_{int}$ is the Hamiltonian of the interaction, determining the strength of light-matter interaction, i.e., the perturbation due to the applied laser field; $H_{r}$ incorporates the relaxation process that is considered time -independent. Using eigenfunction of $H_{0}$ as basis, equation (A1) yields

$\boldsymbol{i\hbar}\dot{\boldsymbol{\rho}_{\boldsymbol{mn}}}\boldsymbol{= i\hbar}\left( \left\langle\boldsymbol{\psi}_{\boldsymbol{m}}\boldsymbol{|}\dot{\boldsymbol{\rho}} \right.\left. \boldsymbol{|}\boldsymbol{\psi}_{\boldsymbol{n}} \right\rangle\right)\boldsymbol{=}\left( \boldsymbol{E}_{\boldsymbol{m}}\boldsymbol{-}\boldsymbol{E}_{\boldsymbol{n}} \right)\boldsymbol{\rho}_{\boldsymbol{mn}}\boldsymbol{+}\left[ \boldsymbol{H}_{\boldsymbol{int}}\boldsymbol{,\rho} \right]_{\boldsymbol{mn}}\boldsymbol{+}\left[ \boldsymbol{H}_{\boldsymbol{r}}\boldsymbol{,\rho} \right]_{\boldsymbol{mn}}$ (A 2)

The relaxation processes are approximated as exponential ^5,7^ . In this case, we can describe a decay matrix by equation (A3),

$\boldsymbol{H}_{\boldsymbol{r}}\mathbf{=}\left[ \begin{matrix} {\frac{\mathbf{1}}{\boldsymbol{\tau}}}_{\boldsymbol{mm}} & \boldsymbol{\cdots} & {\frac{\mathbf{1}}{\boldsymbol{\tau}}}_{\boldsymbol{mn}} \\ \boldsymbol{\vdots} & \boldsymbol{\ddots} & \boldsymbol{\vdots} \\ {\frac{\mathbf{1}}{\boldsymbol{\tau}}}_{\boldsymbol{nm}} & \boldsymbol{\cdots} & {\frac{\mathbf{1}}{\boldsymbol{\tau}}}_{\boldsymbol{nn}} \end{matrix} \right]$ (A 3)

Additionally, we have $\rho_{mn}= {\rho^{*}}_{nm}$, $\tau_{mn}= \tau_{nm}$. The diagonal elements of this matrix, $\tau_{nn}$ , represent the time constants for the relaxation of various states towards their equilibrium values. ^5,8^. we define $\frac{1}{\tau_{mn}} = \gamma_{mn} (m \neq n)$ or the off-diagonal elements, which signify both the rates of population relaxation out of relevant level states and the dephasing caused by collisions between population and its surroundings (elastic interaction).^8^

The decay phenomena of diagonal elements can be described by allowing population to decay from higher-lying levels to lower-lying levels. In such cases, equation (A2) simplifies as follows:^5,7^ :

$\dot{\rho_{mn}}=-i\omega_{nm}\rho_{nm}-\frac{i}{\hbar}\left[ \hat{H}_{int},\hat{\rho} \right]_{nm}- \gamma_{nm}\rho_{nm}$ $\left( n\neq m \right)$

$\dot{\boldsymbol{\rho}_{\boldsymbol{nn}}}\mathbf{=}\frac{\boldsymbol{i}}{\boldsymbol{\hbar}}\left[ {\hat{\boldsymbol{H}}}_{\boldsymbol{int}}\mathbf{,}\hat{\boldsymbol{\rho}} \right]_{\boldsymbol{nn}}\mathbf{+}\sum_{\boldsymbol{E}_{\boldsymbol{m}\boldsymbol{\succ}\boldsymbol{E}_{\boldsymbol{n}}}} \boldsymbol{\Gamma}_{\boldsymbol{nm}}\boldsymbol{\rho}_{\boldsymbol{mm}}\mathbf{-}\sum_{\boldsymbol{E}_{\boldsymbol{m}\boldsymbol{\prec}\boldsymbol{E}_{\boldsymbol{n}}}} \boldsymbol{\Gamma}_{\boldsymbol{mn}}\boldsymbol{\rho}_{\boldsymbol{nn}}$ (A 4)

Here, $\Gamma_{\mathrm{nm}}$ gives the rate in which population decay from level m to level n, and as above, $\gamma_{mn}$ gives the decoherence rate of $\rho_{nm}$ ^5,7^.

Now, we apply the density matrix formalism to the problem of two-level system approximation driven by the electric field of incident light with Gaussian temporal profile. We also consider $\Gamma_{21}=\Gamma_{12}=\frac{1}{T_{1}}$ , and $\gamma_{12}= \gamma_{21}=\frac{1}{T_{2}}$ in order to simplify the derivation of equation (A4) ^5,7^. This yields the following equations:

$$\frac{\partial\rho_{11}}{\partial t}=\frac{i}{2\hbar}\mu_{12}E\left( t \right)\rho_{21}-\frac{i}{2\hbar}\mu_{21}E^{*}\left( t \right)\rho_{12}-\frac{\rho_{11}}{T_{1}}$$

$$\frac{\partial\rho_{12}}{\partial t}=i\omega_{21}\rho_{12}+\frac{i}{2\hbar}\mu_{12}E\left( t \right)(\rho_{22}-\rho_{11})-\frac{\rho_{12}}{T_{2}}$$

$$\frac{\partial\rho_{21}}{\partial t}=-i\omega_{21}\rho_{21}+\frac{i}{2\hbar}\mu_{12}E^{*}\left( t \right)(\rho_{11}-\rho_{22})-\frac{\rho_{21}}{T_{2}}$$

$$\frac{\partial\rho_{22}}{\partial t}=\frac{i}{2\hbar}\mu_{21}E^{*}\left( t \right)\rho_{12}-\frac{i}{2\hbar}\mu_{12}E\left( t \right)\rho_{21}-\frac{\rho_{22}}{T_{1}}$$

(A 5)

If we substitute the electric field expression by equations (A5) as following:

$E\left( t \right) =\left[ A_{p}\cos\left( \omega_{pu}t \right)+A_{pr}\cos\left( \omega_{pr}t \right) \right]exp\left[ -\frac{2 ln2\left( t-t_{0} \right)^{2}}{\tau^{2}} \right]j$= $\left[ A_{p}\cos\left( \omega_{pu}t \right)+A_{pr}\cos\left( \omega_{pr}t \right) \right]e^{j\left[ -\frac{2 ln2\left( t-t_{0} \right)^{2}}{\tau^{2}} \right]}$ (A 6)

Then the four equations are given:

$$\frac{\partial\rho_{11}}{\partial t}=\frac{i}{2\hbar}\mu_{12}\left[ A_{p}\cos\left( \omega_{pu}t \right)+A_{pr}\cos\left( \omega_{pr}t \right) \right]e^{j\left[ -\frac{2 ln2\left( t-t_{0} \right)^{2}}{\tau^{2}} \right]}\rho_{21}-\frac{i}{2\hbar}\mu_{21}\left[ A_{p}\cos\left( \omega_{pu}t \right)+A_{pr}\cos\left( \omega_{pr}t \right) \right]^{*}e^{j\left[ \frac{2 ln2\left( t-t_{0} \right)^{2}}{\tau^{2}} \right]}\rho_{12}-\frac{\rho_{11}}{T_{1}}$$

(A 7)

$$\frac{\boldsymbol{\partial}\boldsymbol{\rho}_{\mathbf{12}}}{\boldsymbol{\partial t}}\mathbf{=}\boldsymbol{i}\boldsymbol{\omega}_{\mathbf{21}}\boldsymbol{\rho}_{\mathbf{12}}\mathbf{+}\frac{\boldsymbol{i}}{\mathbf{2}\boldsymbol{\hbar}}\boldsymbol{\mu}_{\mathbf{12}}\left[ \boldsymbol{A}_{\boldsymbol{p}}\cos\left( \boldsymbol{\omega}_{\boldsymbol{pu}}\boldsymbol{t} \right)\mathbf{+}\boldsymbol{A}_{\boldsymbol{pr}}\cos\left( \boldsymbol{\omega}_{\boldsymbol{pr}}\boldsymbol{t} \right) \right]\boldsymbol{e}^{\boldsymbol{j}\left[ \mathbf{-}\frac{\mathbf{2} \mathbf{ln2}\left( \boldsymbol{t}\mathbf{-}\boldsymbol{t}_{\mathbf{0}} \right)^{\mathbf{2}}}{\boldsymbol{\tau}^{\mathbf{2}}} \right]}\mathbf{(}\boldsymbol{\rho}_{\mathbf{22}}\mathbf{-}\boldsymbol{\rho}_{\mathbf{11}}\mathbf{)-}\frac{\boldsymbol{\rho}_{\mathbf{12}}}{\boldsymbol{T}_{\mathbf{2}}}$$

(A 8)

$$\frac{\partial\rho_{21}}{\partial t}=-i\omega_{21}\rho_{21}+\frac{i}{2\hbar}\mu_{12}\left[ A_{p}\cos\left( \omega_{pu}t \right)+A_{pr}\cos\left( \omega_{pr}t \right) \right]^{*}e^{j\left[ \frac{2 ln2\left( t-t_{0} \right)^{2}}{\tau^{2}} \right]} (\rho_{11}-\rho_{22})-\frac{\rho_{21}}{T_{2}}$$

$\left[ A_{p}\cos\left( \omega_{pu}t \right)+A_{pr}\cos\left( \omega_{pr}t \right) \right]^{*}e^{j\left[ \frac{2 ln2\left( t-t_{0} \right)^{2}}{\tau^{2}} \right]} \left( \rho_{11}-\rho_{22} \right)-\frac{\rho_{21}}{T_{2}}$ (A 9)

$\frac{\boldsymbol{\partial}\boldsymbol{\rho}_{\mathbf{22}}}{\boldsymbol{\partial t}}\mathbf{=}\frac{\boldsymbol{i}}{\mathbf{2}\boldsymbol{\hbar}}\boldsymbol{\mu}_{\mathbf{21}}\left[ \boldsymbol{A}_{\boldsymbol{p}}\cos\left( \boldsymbol{\omega}_{\boldsymbol{pu}}\boldsymbol{t} \right)\mathbf{+}\boldsymbol{A}_{\boldsymbol{pr}}\cos\left( \boldsymbol{\omega}_{\boldsymbol{pr}}\boldsymbol{t} \right) \right]^{\mathbf{*}}\boldsymbol{e}^{\boldsymbol{j}\left[ \frac{\mathbf{2} \mathbf{ln2}\left( \boldsymbol{t}\mathbf{-}\boldsymbol{t}_{\mathbf{0}} \right)^{\mathbf{2}}}{\boldsymbol{\tau}^{\mathbf{2}}} \right]} \boldsymbol{\rho}_{\mathbf{12}}\mathbf{-}\frac{\boldsymbol{i}}{\mathbf{2}\boldsymbol{\hbar}}\boldsymbol{\mu}_{\mathbf{12}}\left[ \boldsymbol{A}_{\boldsymbol{p}}\cos\left( \boldsymbol{\omega}_{\boldsymbol{pu}}\boldsymbol{t} \right)\mathbf{+}\boldsymbol{A}_{\boldsymbol{pr}}\cos\left( \boldsymbol{\omega}_{\boldsymbol{pr}}\boldsymbol{t} \right) \right]\boldsymbol{e}^{\boldsymbol{j}\left[ \mathbf{-}\frac{\mathbf{2} \mathbf{ln2}\left( \boldsymbol{t}\mathbf{-}\boldsymbol{t}_{\mathbf{0}} \right)^{\mathbf{2}}}{\boldsymbol{\tau}^{\mathbf{2}}} \right]}\boldsymbol{\rho}_{\mathbf{21}}\mathbf{-}\frac{\boldsymbol{\rho}_{\mathbf{22}}}{\boldsymbol{T}_{\mathbf{1}}}$ (A 10)

In our study, we used some parameters including $\mu_{nm}$, $\gamma_{nm}$, and $\omega_{nm}$, and they have been achieved for a two-level system (n, m = 1, 2).

The SLG is grown by CVD on copper, is characterised by Raman spectroscopy using a Horiba Labram HR800 spectrometer at 514 nm^9–11^. The 2D peak position (Pos(2D)) and full width at half maximum (FWHM(2D)) are $\sim$ 2684 cm^-1^ and $24\mathrm{cm}^{-1}$ ^9^ , the G peak position (Pos(G)) and full width at half maximum (FWHM(G)) are $\sim$ 1584 cm^-1^ and 13 cm^-1^. The 2D to G peak ratio (A(2D)/A(2G)) is$\sim$ 10, and carrier concentration $\sim$, 6x 10^12^ cm^-1^ was estimated ^9^. From refs^9,11,12^, we estimated electronic absorption $\omega_{nm}\sim583.25 THz (514nm)$. We also calculated the decoherent rates $\gamma_{nm}$, and set to $\gamma_{nm}= 1.824 {ps}^{-1}$ when (m ≠ n) ^9^. We also measured the same value, $\sim2ps$, experimentally (Fig.3c) by time-resolved spectra of SLG, which was obtained by pump-probe imaging, and our results showed a good agreement with the literature^13,14^. From the spectrum, geometry, and sample concentration, we also obtained cross-section of graphene, $\sigma_{nm}\sim2.28\times{10}^{-16} {cm}^{2}$. To predict saturation intensity of SLG, we require to obtain the electric dipole momentum $\mu_{nm}= {\mu^{*}}_{mn}$, when we assumed $\lambda_{pu}=805 nm$, $\lambda_{pr}=1030nm$ and the refractive index of SLG was assumed to be n = 2.58 ^9,15,16^. The electric dipole momentum $\mu_{nm}$ can be obtained from $\sigma_{nm}$ by^8^

$\mu_{nm}= \left( 2\varepsilon_{0}\hbar\gamma_{nm}\sigma_{nm}\lambda_{nm} \right)^{1/2}$ (B1)

Where the decoherent rate is set to $\gamma_{nm}\sim1.824 {ps}^{-1}$ , and the transition wavelength is set to be $\lambda_{nm}\sim514 nm.$ Finally, substituting $\sigma_{nm}$ and $\gamma_{nm}$ into equation (B1), we obtain $\mu_{12}$= 0.196 e nm (for n=1, m=2). As well, according to open Born-Oppenheimer approximation, the electric dipole momentum is mainly determined by the wave function of electrons rather than nuclei^5,8^, thus we considered $\left| \mu_{12} \right|= \left| {\mu_{21}}^{*} \right|=0.196 e.nm$ in our computation. Since a fs laser is used in our theoretical calculations, we assumed different values of $\Gamma= 0.01, 0.1,and 0.11 {ps}^{-1}$. The value of decay rate is $\Gamma=\hbar/\tau$ , where $\tau$is the electron scattering time in SLG^9,15^.

**References**

1. Gong, L. & Wang, H. Suppression of stimulated Raman scattering by an electromagnetically-induced-transparency–like scheme and its application for super-resolution microscopy. *Phys Rev A* **92**, 023828 (2015).

2. Gong, L. & Wang, H. Breaking the diffraction limit by saturation in stimulated-Raman-scattering microscopy: A theoretical study. *Phys Rev A* **90**, 013818 (2014).

3. Brida, D. *et al.* Ultrafast collinear scattering and carrier multiplication in graphene. *Nature Communications* **4**, 1–9 (1AD).

4. Tomadin, A., Brida, D., Cerullo, G., Ferrari, A. C. & Polini, M. Nonequilibrium dynamics of photoexcited electrons in graphene: Collinear scattering, Auger processes, and the impact of screening. *Phys. Rev. B* **88**, 035430 (2013).

5. Weiner, A. M. Ultrafast Optics. (2023) doi:10.1002/9780470473467.

6. Pantell, R. H. & Puthoff, H. E. *Fundamental of Quantum Electronics*. (John Wiley & Sons, 1969).

7. Boyd, R. W. Nonlinear Optics. 1–67 (2008) doi:10.1016/b978-0-12-369470-6.00001-0.

8. Meystre, P. & Sargent, M. Elements of Quantum Optics. 327–349 (2007) doi:10.1007/978-3-540-74211-1_14.

9. Soavi, G. *et al.* Broadband, electrically tunable third-harmonic generation in graphene. *Nature Nanotechnology* **13**, 1–7 (2018).

10. Newson, R. W., Dean, J., Schmidt, B. & Driel, H. M. van. Ultrafast carrier kinetics in exfoliated graphene and thin graphite films. *Opt. Express* **17**, 2326 (2009).

11. Zhao, B., Zhao, J. M. & Zhang, Z. M. Enhancement of near-infrared absorption in graphene with metal gratings. *Appl. Phys. Lett.* **105**, 031905 (2014).

12. Hafez, H. A. *et al.* Terahertz Nonlinear Optics of Graphene: From Saturable Absorption to High‐Harmonics Generation. *Adv. Opt. Mater.* **8**, (2020).

13. Zanini, G., Korobchevskaya, K., Deguchi, T., Diaspro, A. & Bianchini, P. Label-Free Optical Nanoscopy of Single-Layer Graphene. *Acs Nano* **13**, 9673–9681 (2019).

14. Huang, L. *et al.* Ultrafast Transient Absorption Microscopy Studies of Carrier Dynamics in Epitaxial Graphene. *Nano Lett* **10**, 1308–1313 (2010).

15. Vasko, F. T. Saturation of interband absorption in graphene. *Physical Review B* **82**, 245422–6 (2010).

16. Nematpour, A. *et al.* Experimental near infrared absorption enhancement of graphene layers in an optical resonant cavity. *Nanotechnology* **30**, 445201 (2019).
